# Clustering and classification of soybean leaves based on angle features

**DOI:** 10.3389/fpls.2026.1908413

**Published:** 2026-08-28

**Authors:** Haodong Chen, Yanjun Zhang, Haochong Chen, Guangyao Sun, Guanzhou Wu, Fengfeng Liu, Xianfeng Yang, Yuntao Ma, Xingrong Wang, Weiyang Liu

**Affiliations:** 1 College of Agriculture, Tarim University, Alar, China; 2 Key Laboratory of Genetic Improvement and Efficient Production for Specialty Crops in Arid Southern Xinjiang of Xinjiang Corps, Tarim University, Alar, China; 3 Institute of Crop, Gansu Academy of Agricultural Sciences, Lanzhou, Gansu, China; 4 Key Laboratory of Crop Gene Resources and Germplasm Innovation in Northwest Cold and Arid Regions, Ministry of Agriculture and Rural Affairs, Lanzhou, Gansu, China; 5 College of Land Science and Technology China Agricultural University, Beijing, China

**Keywords:** angle feature, K-means, leaf shape classification, machine vision, soybean

## Abstract

**Introduction:**

Leaf shape is a genetically determined crop phenotype, and its accurate classification underpins soybean germplasm assessment and genetic improvement. Manual classification is highly subjective and struggles to distinguish morphologically similar leaves, while mainstream supervised classification demands large labeled datasets and incurs high development costs. Efficient feature frameworks for soybean leaf categorization are still insufficient.

**Methods:**

In this study, 581 biologically replicated terminal leaflets sampled from 194 soybean varieties were analyzed at the single-leaflet level using traditional morphological indices and novel leaf contour angular features. Unsupervised K-means clustering was used to classify soybean leaflet morphological phenotypes; t-SNE was applied exclusively for dimensional reduction visualization, while Welch's ANOVA combined with Games-Howell post-hoc tests was adopted to detect inter-cluster phenotypic differences. Clustering stability and external consistency against manual visual labeling were further quantified via Adjusted Rand Index to comprehensively verify the reliability of grouping outputs.

**Results:**

The results revealed no significant difference in leaflet edge complexity (p = 0.41) between two manually divided leaf groups distinguished by overall leaf outline similarity; these two morphologically similar leaf clusters failed to be fully separated even though the first two principal components accounted for 90.2% of total variance. For K-means clustering, k = 3 achieved better overall performance with a Calinski–Harabasz (CH) index of 395.55, Davies–Bouldin (DB) index of 1.03, and silhouette coefficient (SC) of 0.38, compared with k = 4. Nevertheless, the angular feature attained an F-value of 951.62 in driving sample reallocation across clusters, serving as the core indicator for fine subdivision at k = 4. Under k = 4 clustering, all six morphological indices differed significantly among the four groups (p < 0.05). Additionally, the number of cross-clustered samples increased from 66 to 119 as k rose from 3 to 4, with 96.6% of cross-clustering attributed to the leaflet contour angular feature.

**Discussion:**

This research provides a novel reference and technical support for the automated identification and fine classification of soybean leaf morphology.

## 1 Introduction

The growing global population increases the demand for plant protein and oil. There is an urgent need for efficient identification and exploitation of elite soybean germplasm; accurate leaf phenotypic classification can facilitate germplasm evaluation, accelerate soybean genetic breeding, and support food security. As a dominant protein and oil crop worldwide, soybean yield and quality improvement rely heavily on reliable leaf morphological phenotyping to distinguish varied germplasm resources (Yao et al., 2024). Leaves serve as the core functional organs involved in plant photosynthesis and transpiration. Notably, leaf shape is genetically stable and minimally influenced by environmental fluctuations (Sujata et al., 2011), making it a reliable phenotypic indicator for the identification of different crop varieties. Furthermore, leaf morphology profoundly affects canopy light interception efficiency and regulates crop growth and development (Karami et al., 1972). For example, soybean populations with lanceolate leaves show an improved light radiation interception capacity within the canopy (Bianchi et al., 2020). Given the unique advantages of leaf morphological traits, leaf phenotypic characteristics have been widely adopted for crop variety differentiation in recent studies (Neto and Meyer, 2005; Wäldchen and Mäder, 2018; Yao et al., 2024). Accordingly, establishing a high-accuracy soybean leaf shape classification method is of important theoretical value and practical significance for soybean germplasm evaluation, genetic improvement, and crop phenomics research.

Leaf shape classification is an important aspect of crop phenotyping and germplasm characterization. Two major leaf classification strategies have been established in existing research, each with inherent drawbacks: traditional manual morphological identification (Wäldchen and Mäder, 2018) and computational algorithm-based discrimination (Islam et al., 2025). Manual classification relies on expert visual judgment of leaf contour, venation and texture traits; although easy to implement without specialized hardware, it suffers severe subjectivity and inconsistent scoring criteria for morphologically similar leaf samples. Early soybean germplasm evaluation largely adopted this manual scoring method to categorize leaflet shape types, yet subjective visual ratings often failed to distinguish subtle inter-accession contour differences (Chen and Nelson, 2004).

Driven by advances in digital plant phenotyping, supervised deep learning and unsupervised clustering algorithms such as K-means have been extensively adopted for automated leaf shape categorization (Wang and Shang, 2014; Koklu et al., 2022). For example, Koklu et al. used deep learning networks to extract the deep features of leaves and combined supervised algorithms to achieve the precise classification of grape leaves of multiple varieties (Koklu et al., 2022). Moreover, Islam et al. employed the unsupervised K-means algorithm in combination with leaf shape and color features to complete the species division of tuberous plant leaves (Islam et al., 2025). Recent image-based pipelines have also been applied to soybean leaf phenotyping, enabling high-throughput extraction of geometric shape descriptors from scanned and field RGB leaf images (Yu et al., 2024). Wang et al. further developed multiscale contour matching frameworks to capture leaf outline variations for soybean cultivar discrimination, demonstrating the potential of contour features in variety identification (Wang et al., 2020).

Although these algorithms have significantly improved the objectivity and efficiency of leaf shape classification, there are still shortcomings in the existing research. Supervised algorithms rely on a large number of manually labeled samples and incur high costs for model construction and training, and the labeling process is prone to introducing subjective errors (Wang and Shang, 2014; Elbasi et al., 2024). Whether based on manual classification or algorithm analysis, achieving the accurate identification and effective distinction of leaves with similar shapes remains the core challenge in the current leaf shape classification field (Hewitt and Mahmoud, 2018; Qi et al., 2024). Soybeans feature diverse leaf shapes, with numerous similar types among different soybean varieties (Fattah et al., 2024), which poses great difficulties for precise classification. Systematic investigation of leaf morphological variation across soybean germplasm further confirmed that conventional single geometric indices cannot fully resolve subtle shape overlaps between accessions (Huang et al., 2018b).

Most existing soybean leaf morphology studies merely correlate global leaf geometric indices (e.g., length–width ratio, circularity) with agronomic performance, while few efforts target the establishment of dedicated feature pipelines for fine-scale soybean leaf classification. Critically, prior soybean leaf phenotyping frameworks exclusively adopt holistic shape metrics and fail to extract and utilize local contour angular features, which are capable of capturing subtle boundary variations among morphologically analogous leaflets that cannot be discriminated via conventional global indices. Conventional manual sorting suffers severe subjectivity, whereas supervised deep learning workflows demand massive manually annotated leaf datasets with high development costs; neither strategy satisfies the demand for low-cost, high-throughput germplasm screening. To fill this research gap, this study innovatively integrates traditional global morphological indices with newly defined leaf contour angular descriptors and adopts unsupervised K-means clustering, constructing an efficient, annotation-free system to realize precise fine classification of soybean leaf phenotypes.

To address the above research limitations, this study adopted a diverse panel of 194 soybean germplasm accessions as experimental materials. Beyond classic holistic shape descriptors such as leaf aspect ratio and circularity widely used in prior crop phenotyping, we proposed a set of novel leaf contour angular features. Conventional global morphological metrics only characterize the overall outline of leaflets and fail to capture tiny marginal undulations; by contrast, these local angular variables quantify subtle contour fluctuations along leaf edges, offering a unique fine-scale dimension to discriminate visually similar leaf morphotypes that cannot be separated by traditional indices alone. Combined with unsupervised K-means clustering and multi-statistical verification, this study aimed to three core objectives: first, to extract and validate the discriminative capacity of leaf contour angular features; second, to compare clustering performance between k = 3 and k = 4 schemes and clarify the value of fine leaf subtype subdivision; third, to establish a label-free analytical pipeline for high-throughput, precise soybean leaf morphological classification to support germplasm evaluation and breeding screening.

## 2 Materials and methods

### 2.1 Experimental material

**Figure 1:**
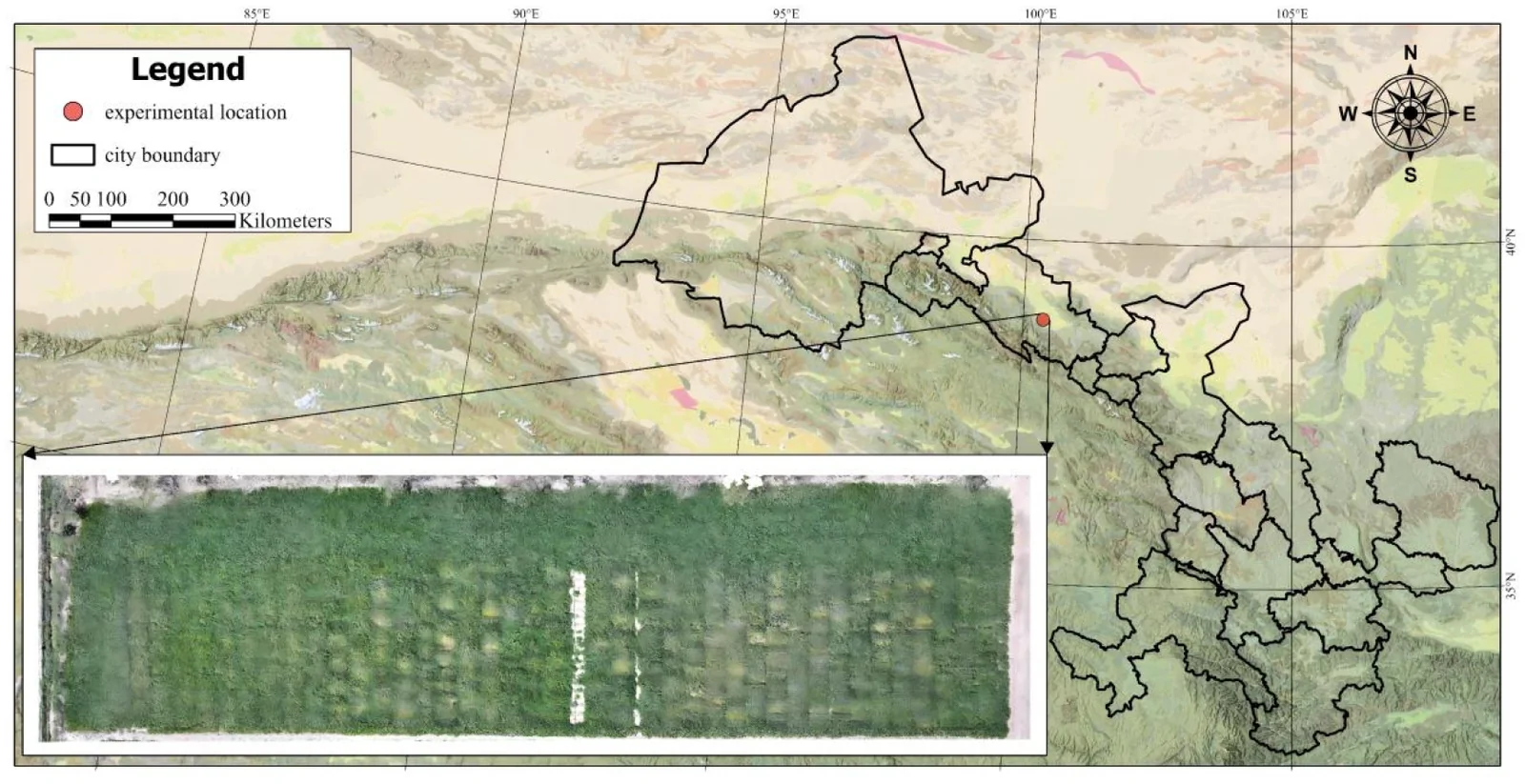
General situation map of study area.

The experimental materials were obtained on August 25, 2025, at the Zhangye Experimental Field of the Gansu Academy of Agricultural Sciences in China (**Figure 1**). On August 25 at the R5 beginning seed stage, intact, disease-free and fully expanded trifoliate leaves were randomly collected from each replicate plot. Only the terminal leaflet of each compound leaf was retained for subsequent morphological measurement, while lateral leaflets were discarded. Immediately after picking, leaf samples were placed into sealed zip-lock bags lined with moist filter paper to maintain leaf turgor and prevent wilting. All samples were transported to the laboratory under low-temperature insulation within 4 h of collection, and all scanning and image acquisition procedures were completed on the same day to avoid dehydration-induced contour deformation. All collected soybeans were cultivated under identical conditions using standardized irrigation and disease control measures. Based on the leaf morphology descriptions of the 194 soybean varieties from germplasm catalogs, the leaflets were manually classified into three categories according to overall visual contour characteristics (leaf roundness, elongation and leaf tip shape).

### 2.2 Leaf image acquisition and preprocessing

**Figure 2:**
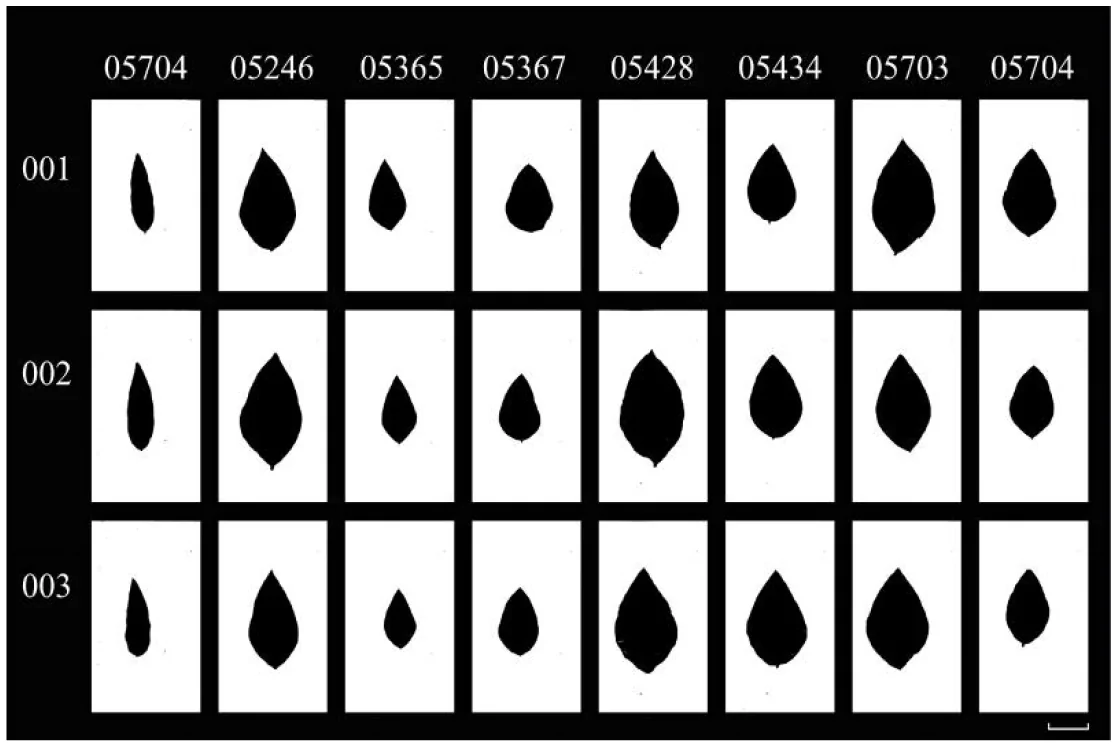
Selected scanned images of soybean leaflets. Each column stands for an individual soybean cultivar with its identification number labeled on the top, and each of the three horizontal rows denotes one replicated apical leaflet sample.

Scanning was performed to capture images of soybean leaflets (**Figure 2**) using the EPSON 700 PHOTO scanner (Seiko Epson Corporation, Suwa, Japan), with a resolution of 6400 dpi. During scanning, pure white A4 paper was utilized as the dedicated background board. Freshly collected soybean leaflets were cleaned of impurities and spread flat at the center of the background board to ensure that the leaflets were free of wrinkles and overlaps. After scanning and imaging, leaflets with severe mechanical damage were removed, yielding a total of 581 scanned images of soybean leaflets.

Image preprocessing for scanned leaflet digital images mainly consisted of foreground-background segmentation, morphological refinement, and target contour extraction, following established phenotyping workflows (Wang et al., 2006; Du et al., 2007). All images were processed via a fully repeatable pipeline implemented in Python OpenCV. Raw RGB scans were first transformed into the HSV color space, and the saturation channel was extracted to distinguish green leaf tissue from soil and shadow backgrounds. Automatic Otsu bimodal threshold segmentation was applied to generate binary masks with two distinct gray levels. This segmentation step precisely isolated the leaf region of interest while removing irrelevant interference from scanning substrates, ambient noise and complex background textures. Manual leaf scanning yielded relatively uniform, simple backgrounds, further facilitating reliable extraction of leaf contour features (Huang et al., 2018a).

Nevertheless, leaf samples unavoidably contained defects including insect holes, which would distort morphological measurements if unaddressed. To minimize such artifact interference, sequential morphological operations were carried out after binarization for noise removal and edge restoration: an opening operation with a 3×3 rectangular kernel eliminated discrete minor background noise, and a subsequent closing operation using a 5×5 rectangular kernel repaired broken leaf margins. Prior to contour detection, flood filling was executed to patch all internal cavities induced by insect feeding damage. During contour screening, only the largest connected component in each binary mask was preserved as the target terminal leaflet contour, whereas tiny spurious contours originating from residual noise were discarded. All filled insect-induced holes were excluded from area and perimeter calculations; all geometric descriptors were computed solely from the complete outer leaf outline after hole remediation, effectively suppressing measurement bias triggered by leaf damage.

### 2.3 Extraction of various leaf shape indicators

#### 2.3.1 Extraction of the leaf shape index

**Figure 3:**
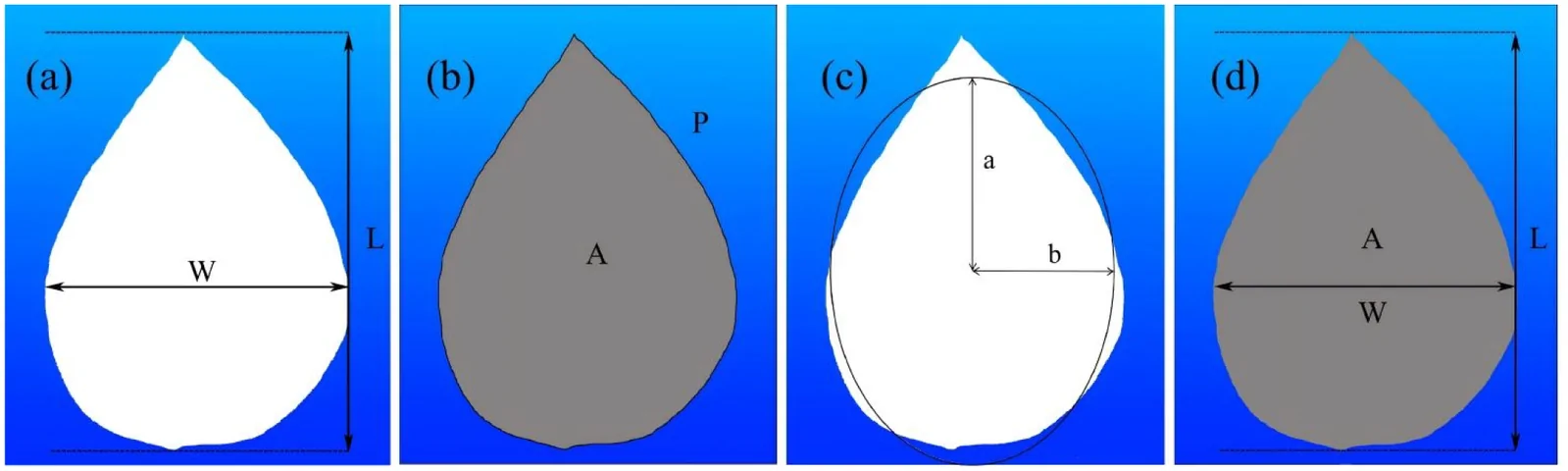
Schematic diagram of the calculation of soybean leaf phenotypic parameters: **(a)** length-to-width ratio; **(b)** roundness and edge complexity; **(c)** eccentricity calculation; **(d)** rectangularity. L represents leaf length; W represents leaf width; A represents leaf area; P represents leaf perimeter; a represents the length of the major axis of the smallest enclosing ellipse; b represents the length of the minor axis of the smallest enclosing ellipse.

Five leaflet phenotypic parameters were extracted from binary images of preprocessed soybean leaves using Python 3.9.0 software and its OpenCV 4.10.0 database. As shown in **Table 1** and **Figure 3**, the basic morphological features included the aspect ratio, eccentricity, circularity, rectangularity, and edge complexity. The minimum bounding rectangle fitted to each leaf contour was a rotated minimum enclosing rectangle rather than a standard axis-aligned rectangle. The ellipse fitting adopted OpenCV’s direct least-squares ellipse fitting algorithm based on all coordinate points of the outer leaf contour, generating the optimal ellipse that matched the overall leaf shape. Prior to calculating all shape and angular descriptors, every segmented leaf contour underwent uniform scale and orientation normalization. All leaf samples were aligned with the long axis of the fitted ellipse to a consistent horizontal orientation, and pixel dimensions were converted to unified physical millimeter units using the fixed reference scale bar placed on each scanning platform, eliminating discrepancies in image magnification and leaf placement angle across samples.

**Table 1:** Soybean leaf phenotypic parameters.

Phenotypic traits	Definition	Acronym	Computational formulas
Aspect Ratio	Ratio of length to width of the leaf’s minimum bounding rectangle	AR	AR = L/W
Eccentricity	Ratio of major axis to minor axis of the leaf’s fitted ellipse	Ec	$Ec=\sqrt{1-{\left(b/a\right)}^{2}}$
Rectangularity	Ratio of leaf area to the area of its minimum bounding rectangle	Re	$RE=A/A_{rect}=A/LW$
Circularity	Measure of how close the leaf’s convex hull is to a circle	Ci	$Ci=4\pi A/P^{2}$
Edge Complexity	Ratio of leaf perimeter to its convex hull perimeter	EC	$EC=P/2\sqrt{\pi A}$

#### 2.3.2 Extracting angle features

**Figure 4:**
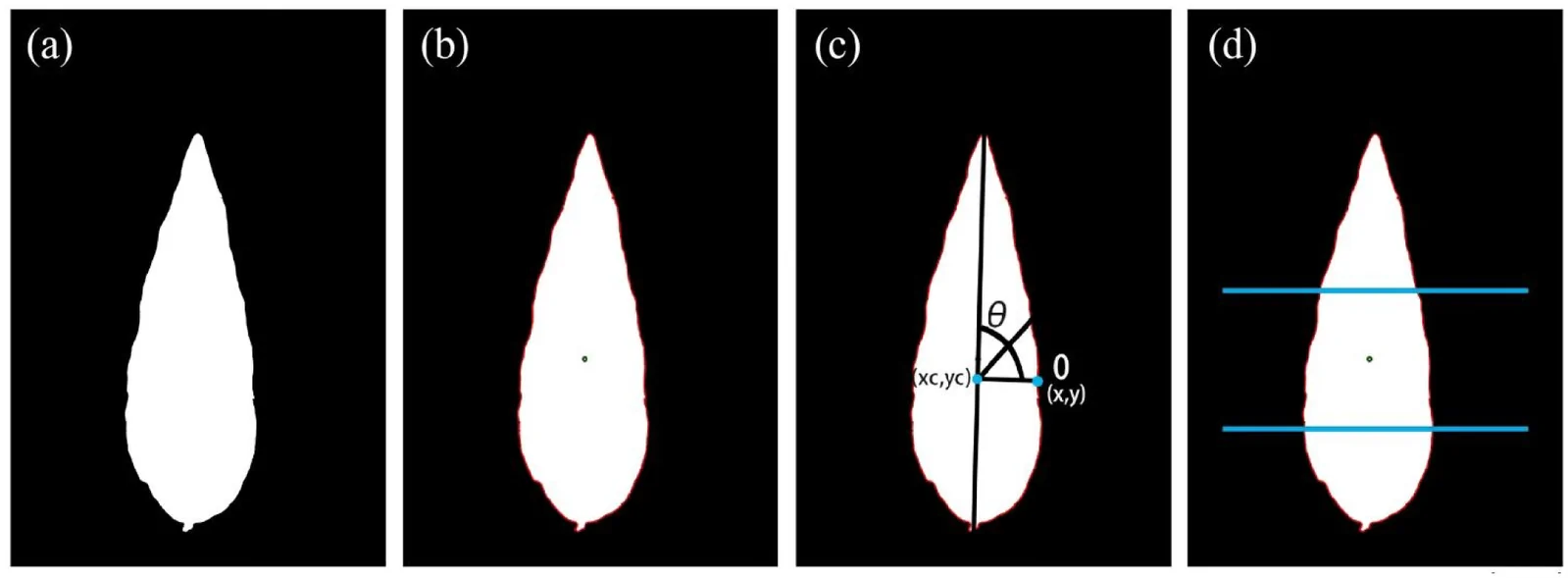
Schematic diagram of angle feature extraction: **(a)** binarized leaflet image; **(b)** global centroid and contour points of the leaflet; **(c)** angle calculation diagram; **(d)** angle feature extraction target area.

Following standardized image preprocessing, pure binary masks of individual leaflets were obtained with accurate isolation of leaf tissues from complex backgrounds. Prior to feature calculation, all leaf contours were subjected to unified orientation normalization to eliminate random rotational deviation caused by manual scanning placement; specifically, each leaflet contour was aligned horizontally based on the long axis of the rotated minimum enclosing rectangle. For angular feature extraction (**Figure 4**), the complete outer contour point set was first acquired from the largest valid connected domain of each standardized leaflet binary image, and the overall contour centroid was subsequently calculated. On this basis, all standardized contour vertices were traversed to derive two types of complementary angular parameters: the polar angle of each contour point relative to the leaflet centroid and the gradient tangent angle determined by the local gradient variation of adjacent contour segments.

To remove biases induced by inconsistent scanning resolution and uneven raw contour-point density, all contour sequences were standardized to a fixed minimum length via cyclic padding and truncation before angular aggregation. These two sets of angular descriptors were computed and fused by averaging corresponding polar and gradient angles to characterize the intrinsic morphological bending traits of leaflet (Equations 1 and 2) margins (Zhang and Lu, 2004; Jähne, 2005). The fused angular metrics possess robust invariance to rotation, reflection, image resolution and contour-point density: pose normalization eliminates arbitrary rotational offsets; angular values reflect relative marginal curvature rather than absolute pixel coordinates, so mirror reflection only reverses vertex order without altering segmental angle statistics; fixed-length contour resampling eliminates fluctuations arising from variable resolution and uneven vertex sampling density.

(1) $$\begin{array}{c} \theta_{i}=mod(\frac{arctan2(y_{i}\;-\;yc,\;x_{i}\;-\;xc)\times 180^{\circ }}{\pi },\;360^{\circ }) \end{array}$$

where *x_i_* and *y_i_* represent the coordinates of the contour points in the pixel coordinate system, while *xc* and *yc* denote the coordinates of the contour centroid in the pixel coordinate system.

(2) $$\alpha =mod(\frac{arctan2(dy,\;dx)\times 180^{\circ }}{\pi },\;360^{\circ })$$

where *dx* and *dy* represent the rates of change of the contour points in different directions in the pixel coordinate system.

After extracting the global contour features of the leaflet images, this study targeted the central area of the leaflet for extraction of the angle features. The extraction process mainly involved calculating the convex hull based on the global contour of the leaflet, followed by determining the main axis of the leaflet length by traversing the vertices of the convex hull, with the middle 1/3 section of the leaflet along the main axis delineated as the target analysis area. Subsequently, utilizing the global angle feature library, the sequences of polar angles and gradient angles corresponding to the contour points falling within the target leaflet section were selected to complete the directional extraction of local angle features. The average of the polar angles and gradient angles was taken to obtain the angle mean, which served as the angle feature of the target area.

### 2.4 Leaflet shape classification based on cluster analysis and statistical analysis

This phenomenon has been characterized as cross-cluster interference in cluster-based statistical frameworks (Leung, 2025), and some scholars have referred to it as contamination. The clustering analysis in this study employed the K-means algorithm. Prior to clustering, Z-score standardization was applied uniformly to all six leaf phenotypic features to eliminate numerical scale differences between conventional morphological indices and angle features, avoiding bias in Euclidean distance calculation caused by inconsistent feature magnitudes. All clustering operations were run in R with a fixed random seed of 123 for reproducibility. Euclidean distance served as the similarity metric, and 25 random centroid initializations were executed to avoid unstable partitions from a single random start; the solution with the lowest WCSS was selected. The algorithm ran with a maximum iteration limit of 1000 and a convergence tolerance of 1×10^-6^. Under this workflow, the number of clusters k is determined, after which k data points are randomly selected from the dataset and utilized as the cluster centers for k clusters. Subsequently, the Euclidean distance from all data points to each initial centroid is calculated, and each data point is assigned to the cluster with the closest centroid. Repeated iterative calculations ensure that each point belongs to the group corresponding to the nearest cluster and the center point no longer changes once convergence is achieved or the maximum iteration count is reached. Finally, the clustering results are obtained (Suyal and Sharma, 2024).

The elbow rule was adopted to determine the optimal number of clusters. This method identifies the optimal k according to the variation trend of within-cluster sum of squares (WCSS) against candidate cluster numbers. For each preset k value, K-means clustering was executed and the corresponding WCSS was calculated via Equation 3 (Jolliffe and Cadima, 2016). A k-WCSS trend curve was plotted, and the elbow inflection point on the curve was used to select the optimal clustering number:

(3) $$\begin{array}{c} WCSS=\sum_{i=1}^{n}\sum_{j=1}^{k}{\left(x_{i}^{\left(j\right)}-c_{i}\right)}^{2} \end{array}$$

where $x_{i}^{\left(j\right)}$ represents the *i*-th sample in the *j*-th cluster, and *c_i_* indicates the center of the *j*-th cluster.

Clustering reproducibility of the K-means algorithm was assessed by running 50 independent replicates with randomly initialized cluster centroids. Pairwise Adjusted Rand Index (ARI) values were calculated between all pairs of clustering partitions generated from repeated runs, and the mean and standard deviation of pairwise ARI were summarized to quantify the stability of grouping results for k = 3 and k = 4.

For external quantitative validation against manual visual classification, a global ARI was further computed to measure consistency between human-annotated morphological labels and the finalized multi-feature k = 4 clustering partition. All cleaned leaf samples with complete manual labels and clustering results were included to construct a full contingency table for one single global ARI calculation. Different from the reproducibility test above, this external ARI only involves one pair of label sets (manual grouping vs. optimal algorithmic clustering) and thus produces a solitary overall consistency metric, rather than replicated pairwise values.

After acquiring stable k = 4 clustering partitions, parametric statistical tests were conducted to compare phenotypic differences across subgroups. Prior to inter-group variance comparison, two core assumptions of parametric analysis were validated for all six leaf morphological traits: normality and homogeneity of variances. Kernel density distribution curves were plotted to assess normality, and all indicators exhibited approximately normal distributions suitable for parametric testing. Homogeneity of variances across the four K-means clusters was further examined via Levene’s test. The results revealed significant variance heterogeneity for five out of six phenotypic indices, violating the prerequisite of conventional one-way ANOVA and LSD-t test.

To obtain unbiased statistical inferences under unequal variance conditions, Welch’s ANOVA with White heteroskedasticity correction was adopted to test overall inter-cluster differences. Pairwise multiple comparisons among subgroups were subsequently conducted using the Games-Howell *post-hoc* test, which is robust to heterogeneous variances and unbalanced sample sizes. The test statistic between cluster *i* and cluster *j* was calculated as (Equation 4) (Games and Howell, 1976):

(4) $$t_{ij}=\frac{{\bar{x}}_{i}-{\bar{x}}_{j}}{\sqrt{\frac{s_{i}^{2}}{n_{i}}+\frac{s_{j}^{2}}{n_{j}}}}$$

where ${\bar{x}}_{i}$, $s_{i}^{2}$ and $n_{i}$ represent the mean, sample variance and sample size of group i, respectively. The degree of freedom for each pairwise comparison was approximated using the Welch-Satterthwaite correction. Studentized range distribution critical values were adopted to adjust p-values for multiple testing, and compact letter displays were generated based on adjusted p-values at α = 0.05.

## 3 Results

### 3.1 Descriptive statistics and correlation analysis

**Figure 5:**
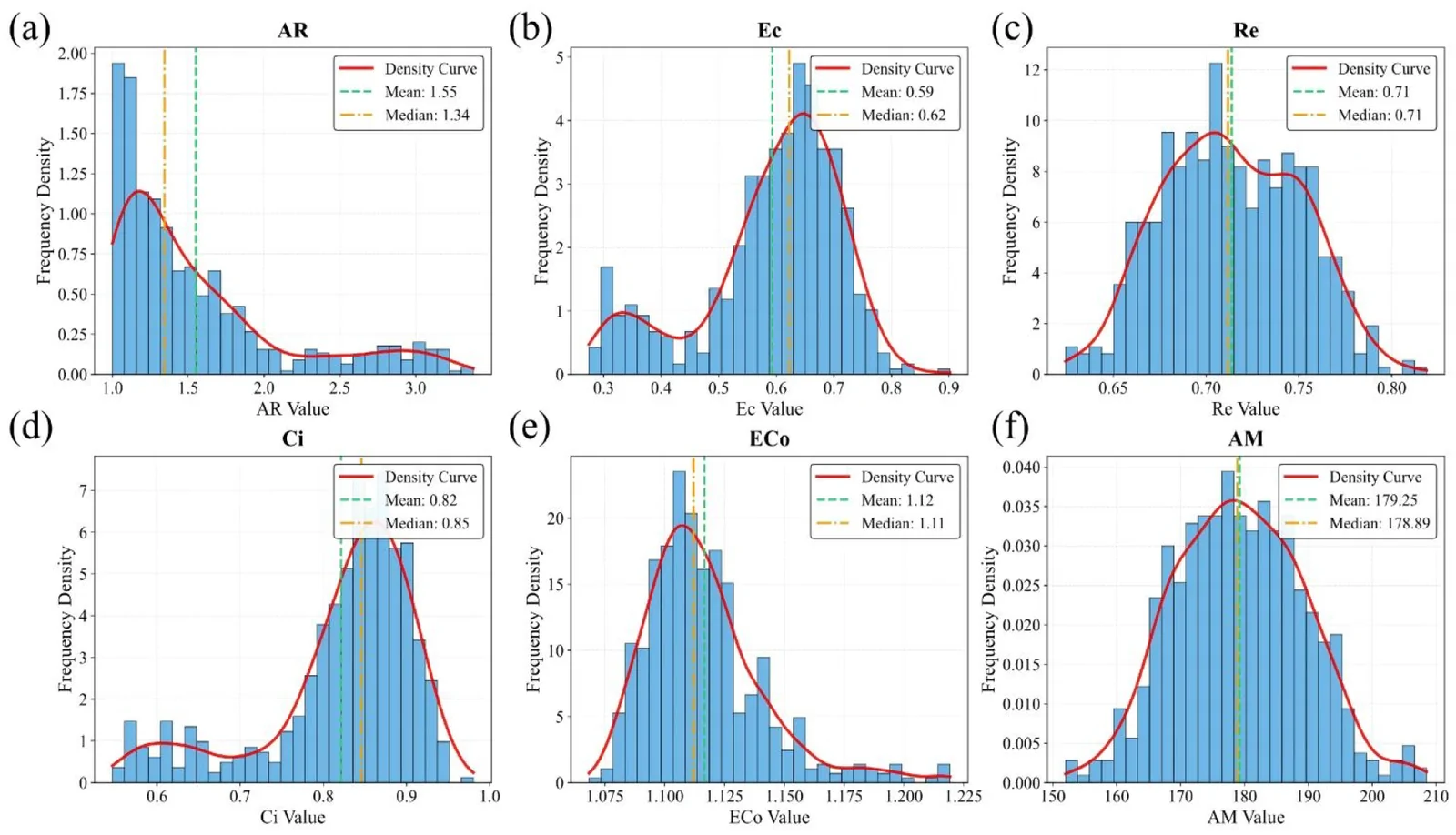
Frequency distribution chart of soybean leaflet morphology indices: **(a)** length-to-width ratio (AR); **(b)** eccentricity (Ec); **(c)** rectangular degree (Re); **(d)** circularity (Ci); **(e)** edge complexity (ECo); **(f)** angle mean (AM).

Frequency distribution and kernel density analyses were conducted on the six soybean leaflet shape indices to reveal the leaflet shape characteristics of the sample population. The distribution of each index is shown in **Figure 5**. Overall, all six shape indices exhibited different degrees of unimodal or nearly unimodal distribution characteristics, and their means closely approximated their medians. This showed that the overall sample distribution was relatively symmetrical, without extreme skewness or abnormal values dominating. The distribution of leaflet eccentricity (Ec), rectangular degree (Re), circularity (Ci), and edge complexity (ECo) was highly concentrated, while the length-to-width ratio (AR) and angle mean (AM) showed a wider distribution range or multi-modal characteristics.

**Table 2** summarizes pairwise correlation coefficients for five global leaflet shape indices and contour angular parameters. Strong linear collinearity existed among leaflet length-width ratio, eccentricity and edge complexity, with correlation coefficients reaching R = 0.95 between length-width ratio and edge complexity, and R = 0.98 between eccentricity and edge complexity. Circularity showed significant positive correlations with eccentricity and edge complexity, with R values of 0.75-0.78. Notably, several morphological indices also exhibited highly negative correlations that form a complex interrelated trait network (**Table 2**). In stark contrast, all correlation coefficients between contour angular features and global shape indices had absolute values below 0.05 and were statistically non-significant. This demonstrates that local angular descriptors are fully orthogonal to conventional holistic leaf metrics, capturing an independent morphological dimension undetectable by traditional shape indicators.

**Table 2:** Correlation analysis of leaflet shape index and angle features.

Index	Aspect ratio	Eccentricity	Rectangularity	Circularity	Edge complexity	Angle-mean
Aspect Ratio	1.00					
Eccentricity	−0.95***	1.00				
Rectangularity	0.11*	−0.13*	1.00			
Circularity	−0.67**	0.78**	−0.11*	1.00		
Edge Complexity	−0.95***	0.98***	−0.13*	0.75**	1.00	
Angle-mean	0.05	−0.04	−0.02	0.00	−0.04	1.00

Asterisks represent significance levels of Pearson correlation coefficients. *p<0.05, **p<0.01, ***p<0.001; no asterisk indicates non-significant correlation(p≥0.05).

### 3.2 Confusion analysis of artificial leaflet classification based on t-SNE dimensionality reduction

**Figure 6:**
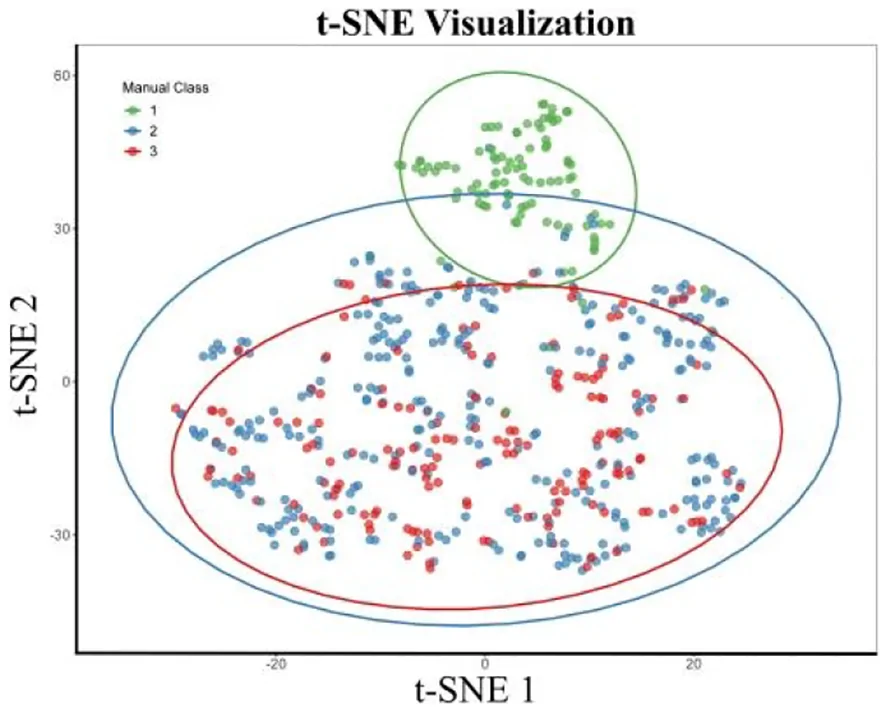
Visualization of the artificial classification results of soybean leaflet phenotypes based on t-SNE dimensionality reduction.

To compare the effectiveness of manual classification and computer clustering, this study visualized the manual classification results utilizing t-SNE dimensionality reduction (**Figure 6**). When manually divided, the three leaflet categories exhibited significant differences in their distribution characteristics in the low-dimensional space. The cluster structure of “green” group leaflets (**Figure 6**) was independent, compactly distributed, and clearly separated from other leaflet categories within the space, indicating that this category of leaves featured stable morphology and high classification consistency. The “red” and “blue” clusters (**Figure 6**) were highly overlapping and had blurred boundaries, showing a continuous transitional distribution, suggesting that their overall morphology was highly similar. The effectiveness of manual classification in this case was significantly limited.

**Figure 7:**
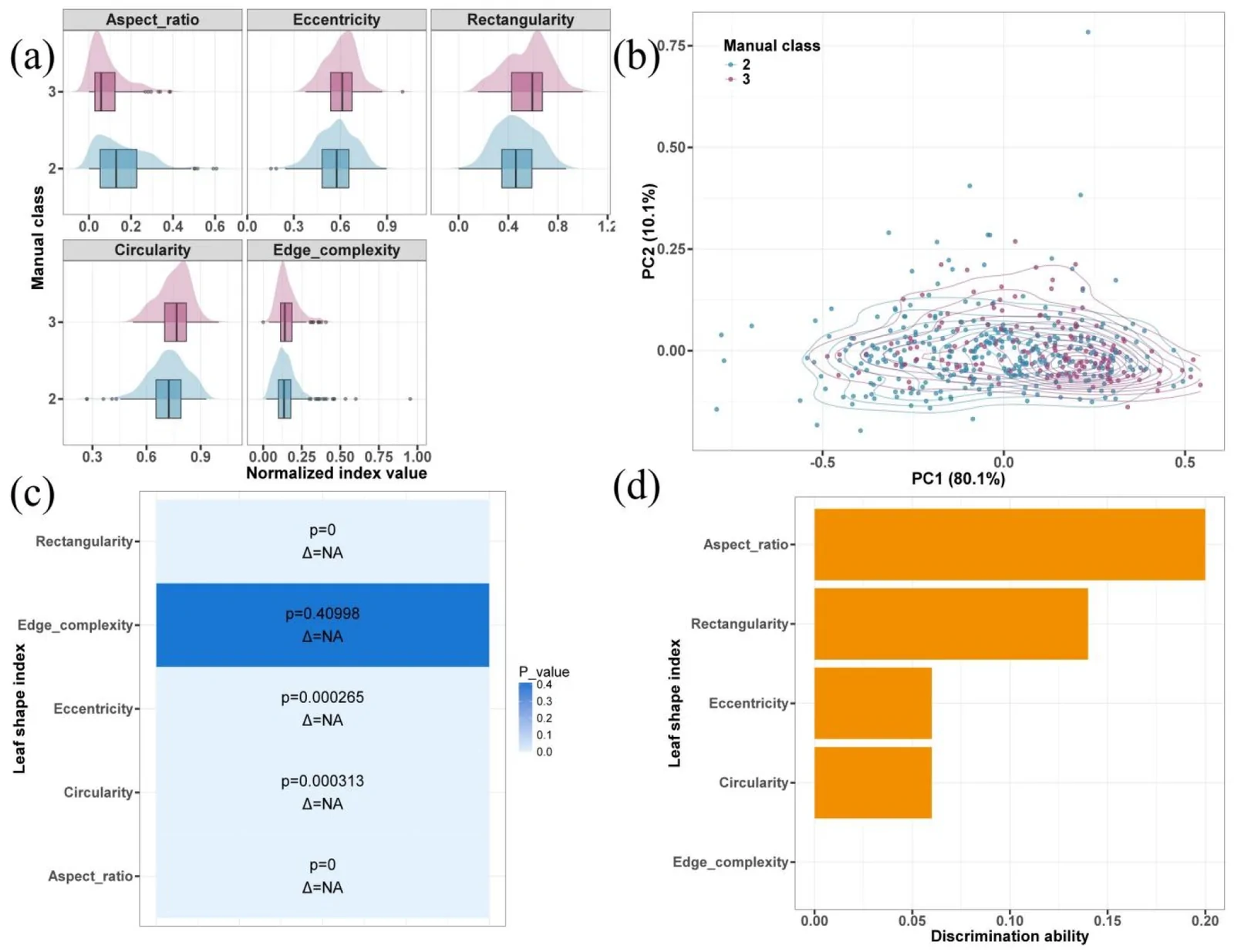
Exploration of the confusion mechanism in the artificial classification of soybean leaflet phenotypes based on t-SNE dimensionality reduction: **(a)** leaflet shape index distribution of the confused categories; **(b)** clustering of soybean leaflet shape indices based on principal component analysis dimensionality reduction; **(c)** significance of differences in leaflet shape indices; **(d)** discrimination ability of the leaflet shape index.

This study further analyzed the internal reasons for the classification confusion by comparing the frequency distribution of leaflet shape indices of the two highly morphologically similar groups (**Figure 7a**) and conducted a significance test. The results showed that the distribution curves of the leaflet shape indices of blue and red groups were highly consistent, with similar peaks and distribution ranges. Combined with the significance analysis (**Figure 7c**), it was observed that except for the edge complexity (p = 0.41), which showed no significant difference (p > 0.05), the other indices were significantly different (p < 0.05). However, the differences were small and could not be clearly distinguished by the naked eye. To verify the morphological similarity and quantify the contributions of different features, this study further validation was confirmed by performing principal component analysis (**Figure 7b**). Although the first two principal components explained 90.2% of the variance, the two sample types were not significantly separated in the core dimension. This confirmed that the overall morphology of blue and red group leaves was highly similar, with a large number of transitional leaf morphologies, posing a challenge for manual classification.

The classification ability of the five leaflet shape indices was ranked (**Figure 7d**). The results showed that edge complexity had the weakest classification ability, followed by circularity and eccentricity, while the aspect ratio the strongest classification ability. Based on the above results, it can be concluded that among the selected leaflet shape indices, edge complexity represents the key weak feature that gives rise to confusion during the manual classification of the two types of leaves.

### 3.3 Leaflet shape classification based on cluster analysis and statistical analysis

#### 3.3.1 Multi-index screening of the cluster number and quantitative assessment of the corresponding clustering effect

The traditional classification of soybean leaflet shapes relies on the manual observation of the leaflet tips, leaflet bases, and leaflet margins. However, this method requires researchers to possess a certain level of expertise in soybeans, and the classification criteria lack clarity.

**Figure 8:**
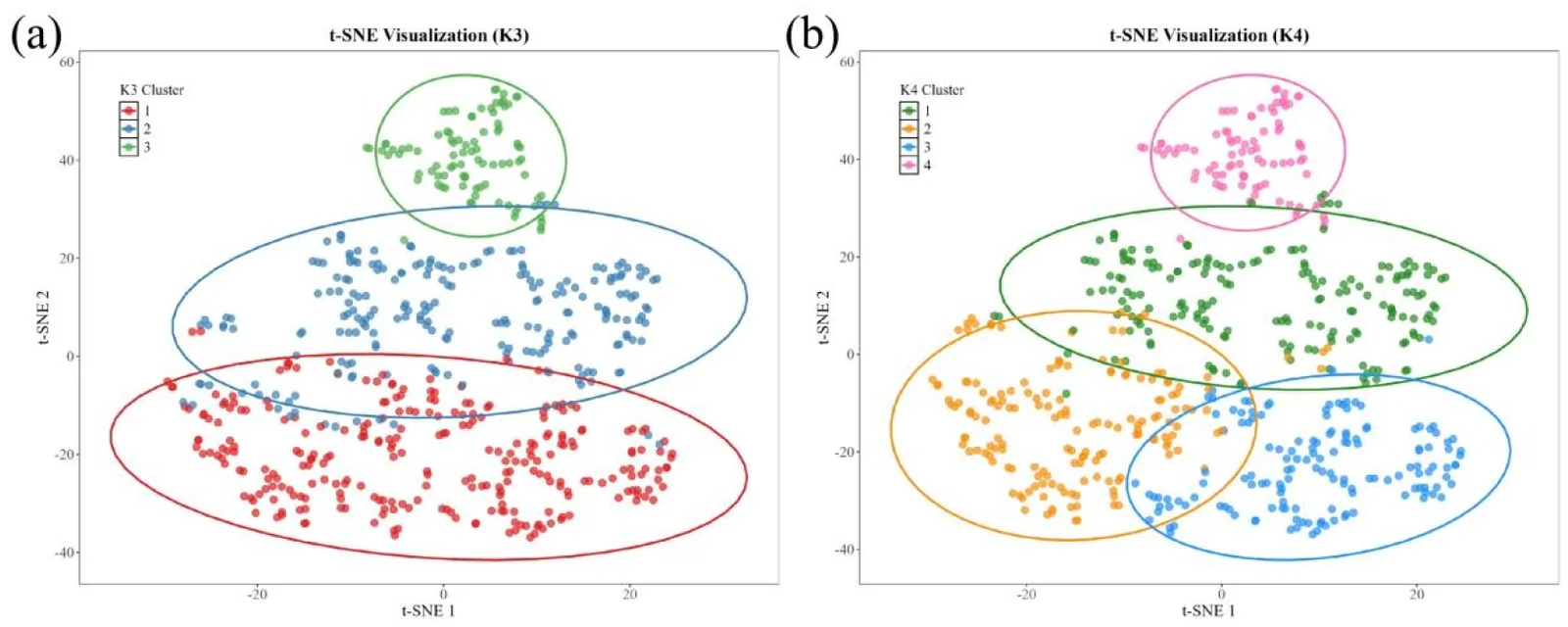
Visualization of soybean leaflet phenotypic clustering based on t-SNE: **(a)** phenotypic clustering for k = 3; **(b)** phenotypic clustering for k = 4.

Soybean leaflet morphological phenotypes were objectively classified via the K-means algorithm using combined contour indices and leaf spatial angle features. The clustering outcomes for leaf samples from 194 soybean genotypes are visualized in **Figure 8**. The elbow method indicated that soybean leaf morphology could be reasonably partitioned into three or four distinct categories. In **Figure 8**, color-coded ellipses denote separate clustering subgroups. Slight overlapping regions exist between clusters, as several soybean genotypes exhibit subtle transitional leaf phenotypic characteristics that blur inter-group boundaries. Under the optimal four-cluster solution (k = 4), the sample sizes varied across subgroups. Cluster 1 contained the largest number of leaflet samples (182, 31.33%), followed by Cluster 2 (163, 28.06%) and Cluster 3 (149, 25.65%). Cluster 4 held the fewest specimens (87, 14.97%). The total number of valid leaflet samples used for clustering analysis was 581. Although Cluster 4 had a relatively smaller sample volume, the sample size remained sufficient to support reliable inter-cluster statistical comparison via Welch’s ANOVA and Games-Howell *post-hoc* tests.

Clustering reproducibility was quantified using 50 independent K-means replicates with distinct random initial centroids, and pairwise ARI values were computed to evaluate partition consistency. Moderate reproducibility was observed for both clustering schemes: the mean pairwise ARI reached 0.6605 (SD = 0.2738) for k = 3, and rose to 0.7593 (SD = 0.2062) for k = 4, demonstrating superior stability of the four-category partition.

A global ARI covering all valid leaf samples was further calculated to quantify consistency between manual visual grouping and the multi-feature k = 4 clustering output, yielding a value of 0.1732 and indicating weak overall agreement between human visual judgment and automated partitioning. This large discrepancy arises from fundamental differences in feature utilization: manual visual classification exclusively relies on two-dimensional leaf contour geometry and cannot capture leaf spatial angle information. The angle feature acts as the dominant discriminative variable that separates visually ambiguous leaf subgroups, which cannot be distinguished by contour-only human observation.

#### 3.3.2 Analysis of the basis and rationality of different cluster number selections

**Figure 9:**
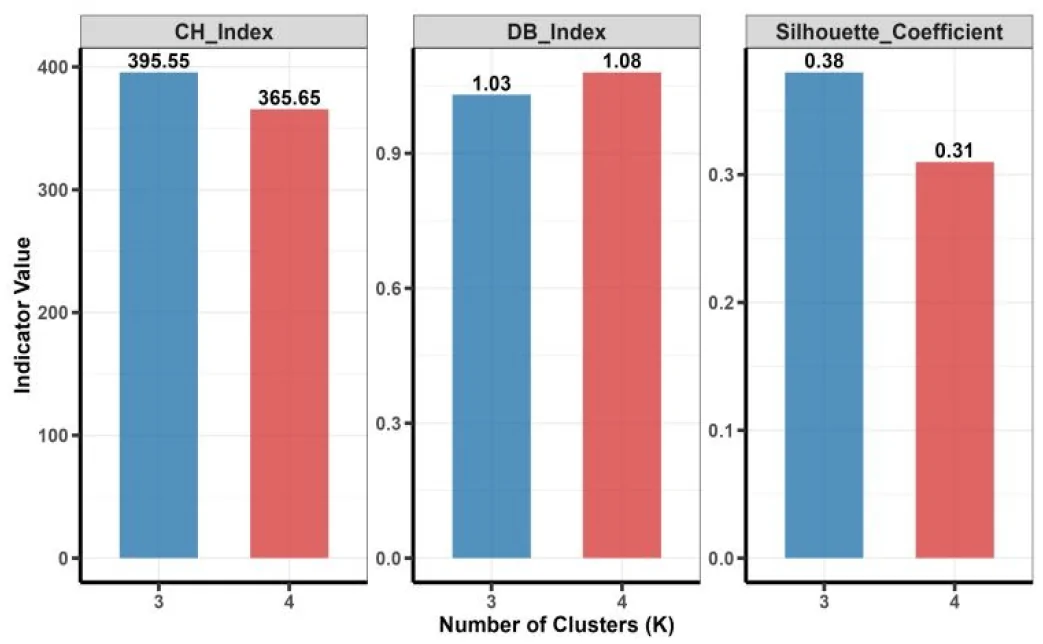
Cluster quality comparison chart.

The quantitative validity of the clustering results obtained using k = 3 and k = 4 was evaluated using the Calinski–Harabasz (CH) index, Davies–Bouldin (DB) index, and silhouette coefficient (**Figure 9**). The results showed that when k increased from 3 to 4, the CH index decreased from 395.55 to 365.65, revealing a decline in the comprehensive performance of inter-class dispersion and intra-class compactness. The DB index rose from 1.03 to 1.08, suggesting an increase in the degree of overlap between clusters and reduced clustering separability. The silhouette coefficient decreased from 0.38 to 0.31. Overall, the clustering results for k = 3 were superior to those for k = 4 in terms of structural compactness and category discrimination.

Although k = 3 obtained better scores across CH, DB and silhouette coefficient, this study prioritized the interpretation of k = 4 clustering with robust statistical and biological justifications. Statistically, contour angular features had an F-value of 951.62 for inter-group discrimination, capturing subtle leaf phenotypic variance neglected by global clustering metrics. Increasing cluster number to k = 4 separated ambiguous overlapping leaf samples that could not be differentiated at k = 3, and 96.6% of cross-clustered sample shifts were attributed to angular traits. Biologically, all six leaf indices showed significant differences among the four subgroups (p < 0.05), enabling fine identification of cryptic leaf morphotypes within diverse soybean germplasm. Such refined partitioning supplies detailed phenotypic stratification data for germplasm evaluation and genetic analysis, whereas the coarser k = 3 grouping loses this subtle morphological variation information. For these reasons, the k = 4 partition was emphasized despite inferior overall clustering validity indices.

**Figure 10:**
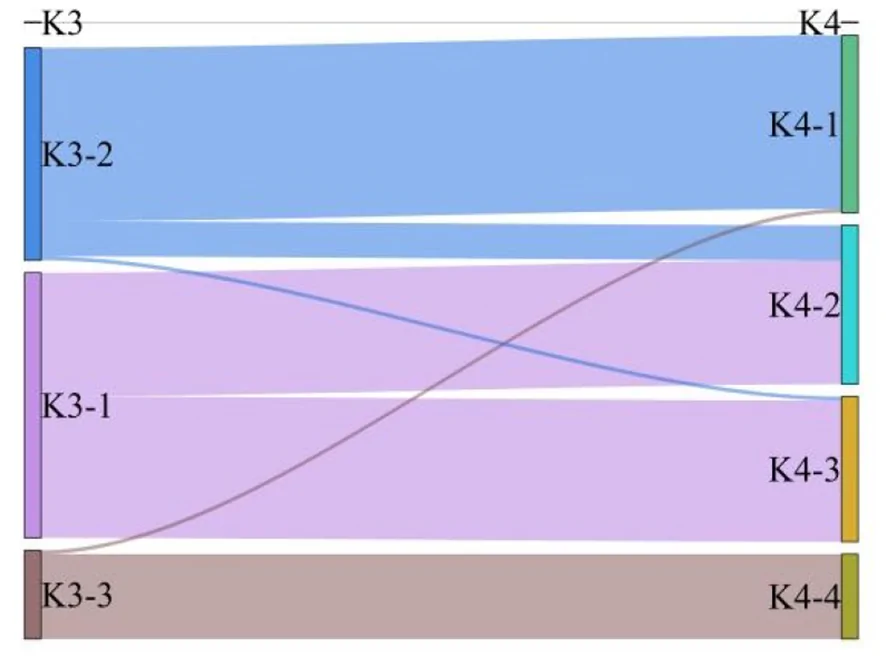
Sankey diagram of sample flow for k = 3 through k = 4.

To further clarify the distribution patterns of the samples under different cluster numbers, this study employed a Sankey diagram to visually present the sample redistribution process from k = 3 to k = 4 (**Figure 10**). The results showed that the samples in the original K3–1 cluster were redistributed to the K4–2 and K4–3 clusters, while only a small number of samples in K3–2 and K3–3 were transferred. Combined with the aforementioned evaluation results for clustering validity, it can be concluded that although the overall clustering quality of k = 3 is higher, k = 4 is not a meaningless splitting of cluster structures but a refined subdivision of the cluster structure with K3–1 as the main object. This reflects the identification of further leaflet morphology subtypes with enhanced clustering granularity.

**Figure 11:**
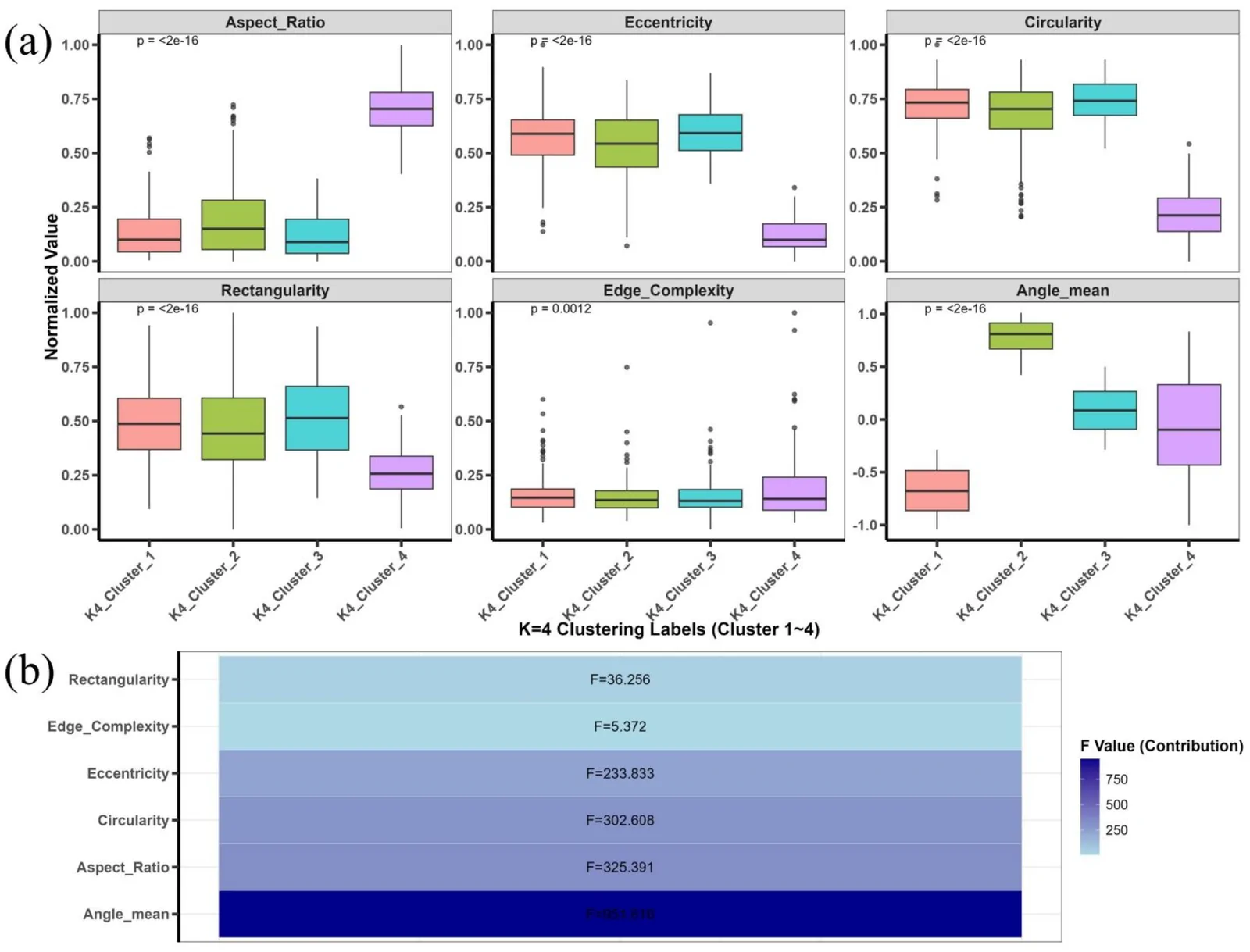
Cluster segmentation effect analysis: **(a)** box plot comparing the discrimination capabilities of different morphological features; **(b)** heatmap of feature importance for different morphological features.

To analyze the key characteristics of the k = 3 to k = 4 cluster restructuring, this study systematically analyzed the morphological indicators using box plots and feature importance heatmaps (**Figure 11**). The box plot results indicated that among the six features (including aspect ratio, eccentricity, and circularity), the angular feature exhibited the most significant distribution differences among the k = 4 clusters, making it the core morphological indicator for distinguishing newly formed subclusters. Edge complexity exhibited a relatively weak distinguishing ability, while the overlapping degrees among the other features were high and the distinguishing effect was limited. The feature importance heatmap further quantified the contribution degree of each feature to the clustering reconstruction. Among them, the importance of the angular feature was 951.616, significantly higher than other indicators, confirming the angular feature as the core determinant factor driving the redistribution of samples from k = 3 to k = 4.

#### 3.3.3 Analysis of the basis and rationality of different cluster number selections

Based on the k = 4 clustering results, this study conducted Welch’s ANOVA and Games–Howell *post-hoc* tests on six morphological phenotypic indicators in four groups of soybean leaves (**Table 3**). Significant pairwise inter-group differences were detected for five of the six leaf morphological traits, while edge complexity showed no significant difference between Group 2 and Group 4. Group 3 achieved the highest levels in eccentricity (0.69 ± 0.004c), rectangular degree (0.75 ± 0.002c), circularity (0.89 ± 0.002c), and mean angle (259.02 ± 5.153c), while the aspect ratio (1.15 ± 0.01c) and edge complexity (1.11 ± 0.001c) were the lowest. This indicated that the overall morphology of the leaves in this group was closer to a circle, with a regular outline and smooth edges. Group 4 exhibited the highest aspect ratio (2.81 ± 0.037d) and edge complexity (1.14 ± 0.006b) and the lowest eccentricity (0.35 ± 0.005d), rectangular degree (0.67 ± 0.003d), and circularity (0.62 ± 0.005d). The leaflet morphology was more elongated, and the edges were irregular. In Groups 1 and 2, most of the phenotypic indicators were at intermediate levels. Among them, Group 2 had a significantly lower mean angle (85.43 ± 4.516b) than the other three groups, exhibiting a unique leaflet spatial angle characteristic. For edge complexity, Group 2 and Group 4 shared the same significance letter “b”, indicating no statistical difference between these two clusters on this single indicator.

**Table 3:** Statistical analysis of phenotypic differences between soybean leaflet groups.

Group	Phenotypic traits					
	Aspect ratio	Eccentricity	Rectangularity	Circularity	Edge complexity	Angle-mean
Group 1	1.65 ± 0.021a	0.56 ± 0.004a	0.69 ± 0.001a	0.81 ± 0.003a	1.12 ± 0.002c	199.03 ± 7.204a
Group 2	1.22 ± 0.012b	0.66 ± 0.004b	0.73 ± 0.002b	0.87 ± 0.002b	1.13 ± 0.003b	85.43 ± 4.516b
Group 3	1.15 ± 0.01c	0.69 ± 0.004c	0.75 ± 0.002c	0.89 ± 0.002c	1.11 ± 0.001c	259.02 ± 5.153c
Group 4	2.81 ± 0.037d	0.35 ± 0.005d	0.67 ± 0.003d	0.62 ± 0.005d	1.14 ± 0.006b	195.66 ± 11.322b

Statistical comparisons were performed using Welch’s ANOVA followed by Games-Howell *post-hoc* tests. Different lowercase letters within the same column indicate significant differences between groups at p < 0.05; identical lowercase letters denote non-significant differences.

**Figure 12:**
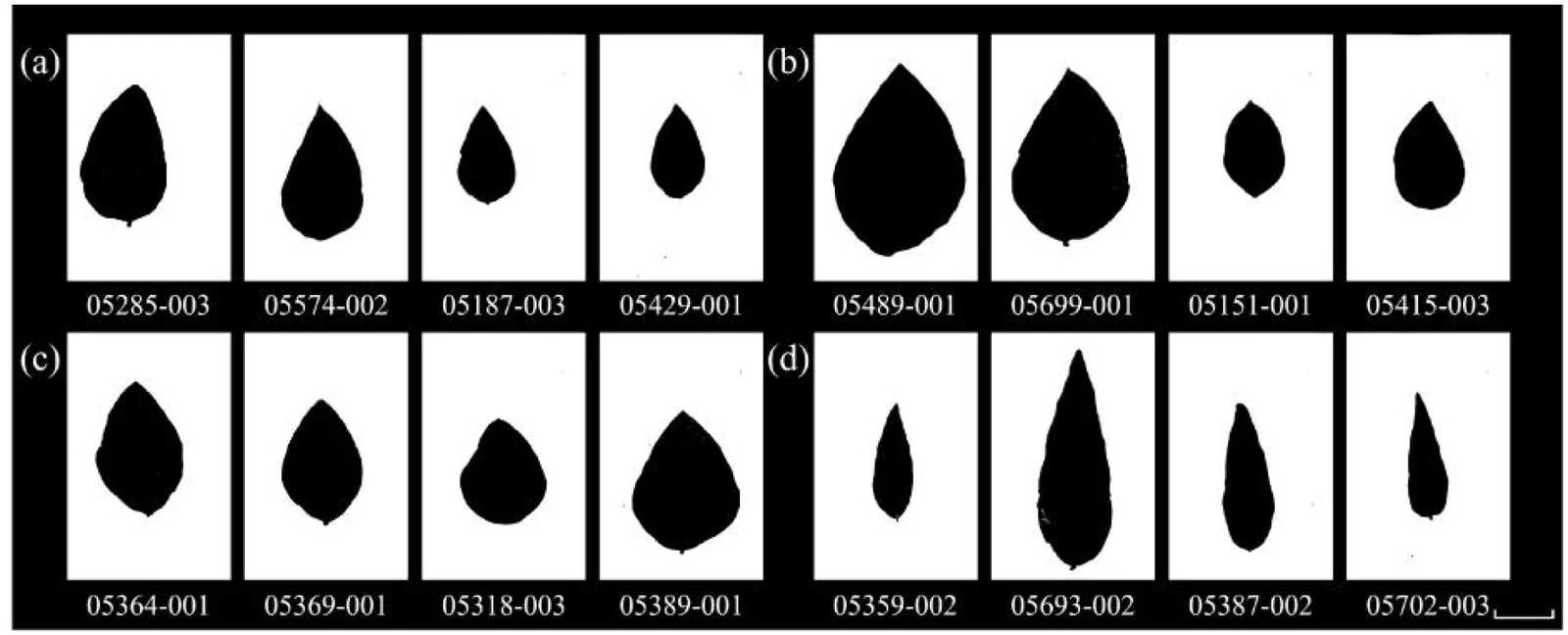
Silhouettes of typical soybean leaves: **(a)** Group 1; **(b)** Group 2; **(c)** Group 3; **(d)** Group 4.

Based on the results of cluster analysis and statistical analysis, four representative soybean leaves were selected from each of the four groups for display, as shown in **Figure 12**. From an intuitive perspective, the leaves in Group 4 can be clearly distinguished from the other three groups by the naked eye, while the external shapes of Groups 1, 2, and 3 are relatively similar, potentially leading to confusion when observed with the naked eye. Nevertheless, the above statistical analysis results indicate that computer clustering based on morphological phenotypic indicators can still effectively distinguish these three groups of easily confused leaves.

### 3.4 Cross-clustering and its driving mechanism

**Figure 13:**
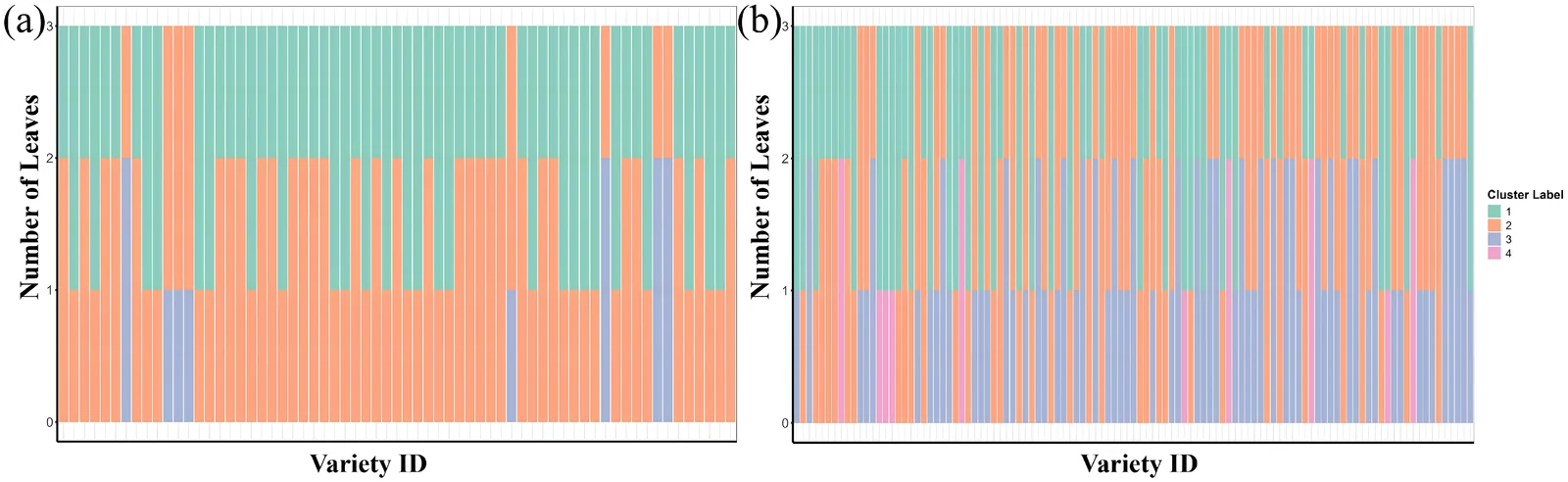
Cross-clustering sample stacking plots: **(a)** for k = 3 clusters; **(b)** for k = 4 clusters.

During the unsupervised clustering analysis of soybean leaf phenotypes, a significant cross-clustering phenomenon was observed (**Figure 13**). Different leaflet samples of the same variety were assigned to different clusters during the clustering process. Some scholars have referred to this phenomenon as contamination (Hudgens and Halloran, 2008; Staples et al., 2015). In this study, when the number of clusters increased from k = 3 to k = 4, the number of cross-cluster samples rose from 66 to 119. Based on a systematic analysis of leaflet shape indices and angle features, we conclude that the angle feature is the key factor driving this phenomenon.

From the perspective of the feature responses resulting from the increase in the number of clusters, the cluster structure reconfiguration from k = 3 to k = 4 is mainly driven by the angular features, indicating that angular features exhibit a higher degree of discriminatory ability in distinguishing the phenotypic subtypes of leaves, with the potential to capture subtle phenotypic differences that traditional leaflet shape indices cannot reflect. When the number of clusters increases, the algorithm tends to subdivide samples based on the variation of angular features, thereby causing the leaves of the same variety to be classified into different clusters.

**Figure 14:**
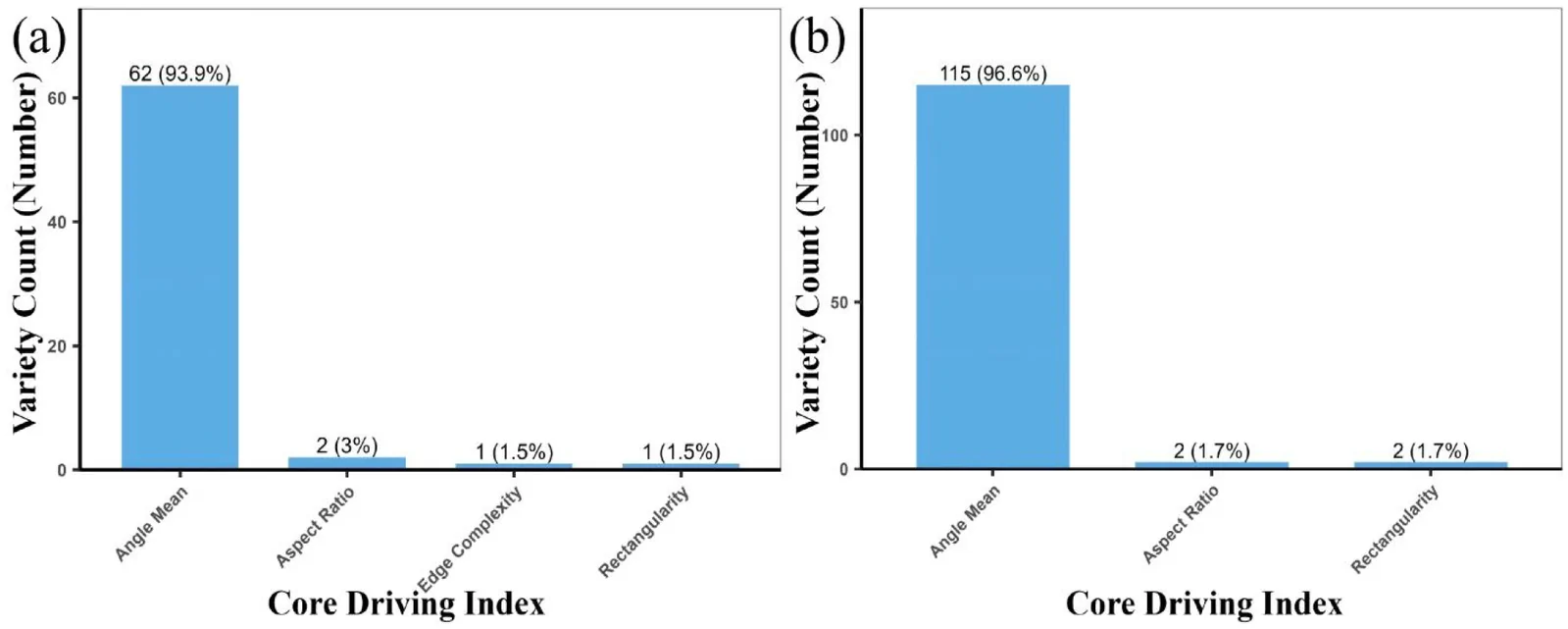
Frequency graphs of the occurrence frequency of morphological parameters in different clustering groups in the cross-clustering phenomenon: **(a)** core driving index of cross-clustering varieties when the clustering quantity k = 3; **(b)** core driving index of cross-clustering varieties when the clustering quantity k = 4.

To explore the role of leaflet shape index and angle characteristics in the cross-clustering phenomenon, this study employed the bias–variance trade-off measure (Karami et al., 1972) to record the frequency of occurrence of different morphological parameters to quantify phenotypic heterogeneity within varieties (**Figure 14**). At the leaf sample level, the deviation counts of angle features reached 62 under k=3 clustering and 115 under k=4 clustering, numerically exceeding the deviation frequencies of all conventional leaf shape indices. A Chi-squared goodness-of-fit test coupled with Bonferroni-adjusted pairwise comparisons was implemented to statistically quantify inter-trait disparities in intra-cultivar phenotypic fluctuation, with analyses restricted exclusively to varieties exhibiting cross-clustering behavior. All pairwise statistical outputs are summarized in **Table 4**.

**Table 4:** Pairwise Chi-squared comparisons between angle-mean and conventional leaf morphological indices (Bonferroni corrected α = 0.01).

Compared trait	Deviation frequency of angle-mean	Deviation frequency of compared trait	χ^2^ value	Raw p-value	Bonferroni adjusted p	Significance
Aspect ratio	24	38	3.97	0.0464	0.2318	ns
Edge complexity	24	27	0.11	0.7452	3.7258	ns
Rectangularity	24	8	8.39	0.0038	0.0189	ns
Eccentricity	24	2	19.53	<0.001	<0.001	**
Circularity	24	0	25.08	<0.001	<0.001	**

ns, no significant difference (adjusted p > 0.01); **highly significant difference (adjusted p < 0.01). Deviation frequency refers to the number of varieties where intra-variety CV exceeded the 75th percentile.

Pairwise Chi-squared comparisons with Bonferroni correction (α = 0.01) in **Table 4** revealed that the angle-mean feature had markedly higher intra-variety deviation frequency than eccentricity and circularity, showing highly significant inter-trait differences (adjusted p < 0.001); by contrast, no significant discrepancies were observed when angle-mean was compared with aspect ratio, edge complexity and rectangularity (all Bonferroni-adjusted p > 0.01), which collectively verifies that angular contour features possess stronger within-variety morphological heterogeneity than most traditional leaf morphological indicators, and this prominent phenotypic variation of angular traits serves as the dominant factor driving sample cross-clustering across K-means partitions.

## 4 Discussion

Current research on plant leaflet classification predominantly employs methodologies such as multi-scale shape representation, deep learning-based feature extraction, or two-dimensional contour landmark algorithms (Kadir et al., 2013; Mouine et al., 2013; Zhong et al., 2025). While these approaches exhibit distinct emphases in terms of feature selection or model complexity, they often overlook subtle geometric characteristics at the leaflet margin, such as spatial angular attributes (Sachar and Kumar, 2021). Classic contour feature extraction algorithms including Fourier descriptors and shape context represent widely used alternatives for plant organ shape quantification, yet they have inherent defects when applied to fine discrimination of soybean leaflets. Fourier descriptors convert complete leaf outlines into frequency-domain coefficients, which smooth high-frequency tiny marginal angular fluctuations during transformation and discard the local serration deflection signals that dominate intra-variety leaf divergence; they only deliver global shape information without capturing spatially localized edge posture differences. Shape context builds log-polar histograms for contour landmarks to describe surrounding point distributions, but it merely records relative spatial distances rather than explicitly calculating marginal deflection angles, failing to quantify the subtle directional variations of leaf serrations. Neither of these two mainstream contour algorithms can independently extract explicit local angular metrics of leaf margins.

Low correlation coefficients and insignificant statistical outcomes between the newly-proposed angle mean and conventional global-shape indices demonstrate the orthogonality between the two sets of morphological parameters. Traditional shape descriptors capture the overall outline properties of leaflets, whereas angle-mean targets local geometric undulation along leaf margins. The weak linear association does not indicate poor sensitivity of the angular indicator to leaf-shape variation. On the contrary, it proves that marginal angular information corresponds to an independent morphological dimension which cannot be covered by common global shape indices.

Raw values of the six leaf morphology features cover vastly different numerical ranges. The five conventional shape indices mostly vary from 0 to 1, whereas angle mean generates considerably larger raw values. Z-score standardization was adopted to remove the bias caused by inconsistent feature scales and grant equal initial weight to every morphological dimension in K-means clustering. Owing to its large magnitude raw data scale, the angle mean feature would otherwise dominate distance computation and weaken the contribution from global shape traits. Standardization guarantees that local marginal angular property and conventional global shape attributes participate equally in cluster partitioning, which matches our research objective of exploring independent leaf shape dimensions.

In sharp contrast, our framework integrates conventional global shape indices with a newly defined mean marginal angle feature, which provides an orthogonal fine-scale dimension unavailable in Fourier descriptors or shape context. This framework effectively enhances the recognition capability for morphologically similar leaves, addresses the limitations of traditional indices in capturing fine-grained morphological variations.

The results of manual classification show that some categories are highly overlapped and have blurred boundaries in the t-SNE space, with obvious visual confusion. Morphological comparison reveals that although partial leaf shape indicators differ between these overlapping categories, their variation amplitudes are narrow, and a large number of transitional intermediate samples exist, leading to difficulty in visual discrimination by naked eye. Edge complexity exhibits weak inter-group discriminative capacity and serves as the main factor causing visual confusion during manual identification. In contrast, computer clustering integrated with multiple morphological features can clearly separate these easily confused leaf subgroups, which proves that the multi-index classification system established in this study has better stability and objectivity. Furthermore, this result indirectly confirms that manual classification and computerized phenotype clustering should adopt distinct evaluation criteria (Sokolova and Lapalme, 2009).

The consistency between manual visual classification and automated clustering is further quantified using a global ARI metric covering all leaf specimens. The low overall ARI value of 0.1732 quantitatively demonstrates the inherent limitation of contour-dependent manual identification: human observers cannot capture subtle spatial posture differences reflected by the mean angle trait, leading to blurred inter-group boundaries and abundant transitional leaf samples that cannot be finely distinguished by naked eyes alone. In contrast, multi-feature clustering integrating both contour geometry and leaf spatial angle information enables fine-grained morphological subdivision unavailable through manual observation. Combined with the high reproducibility of the k = 4 clustering scheme (mean ARI = 0.7593 across repeated algorithm runs) and significant intra-variety variability of angular features verified by Chi-squared tests (**Table 4**), this multi-layer quantitative evidence jointly confirms the robustness and unique discriminative value of the proposed phenotyping classification system.

Statistical comparisons based on Welch’s ANOVA and Games-Howell *post-hoc* tests revealed significant pairwise differences across the four clusters for five of the six leaf phenotypic indicators. Notably, Group 2 and Group 4 showed statistically indistinguishable edge complexity (sharing the same letter “b”), while significant differences existed between the two clusters for all other five leaf morphological features. This result reveals that edge complexity, describing the overall contour winding degree of leaflets, provides limited discriminatory information to separate these two subgroups. However, the integrated multidimensional morphological signature combining aspect ratio, eccentricity, rectangularity, circularity and angle-mean captures distinct geometric properties, enabling reliable separation of Group 2 and Group 4 in unsupervised clustering. It further demonstrates that single shape trait is insufficient to characterize leaflet morphological variation; joint utilization of multiple complementary geometric indicators is essential for fine-grained leaf phenotyping classification.

Among all four clusters, Group 4 can be directly distinguished by the naked eye, while the other three groups have similar morphologies and are easily confused but can be clearly distinguished after clustering, thus demonstrating the application value of this multi-feature classification method in actual phenotypic identification.

Cluster analysis revealed significant cross-clustering in which multiple leaves from the same variety were allocated to different clusters. As the number of clusters increased from k = 3 to k = 4, the proportion of cross-clustered samples rose. This phenomenon indirectly corroborates the substantial variability in leaf morphology within the same species (Hewitt and Mahmoud, 2018). According to cluster validity indices, the intra-cluster compactness and inter-cluster separation at k = 3 are generally superior to those at k = 4. However, Sankey diagram analysis indicates that k = 4 does not represent arbitrary partitioning but rather a meaningful refinement of the original cluster structure (Jika, 2025). This outcome confirms that cross-clustering is not a result of misclassification (Coroenne et al., 2026). There are two plausible explanations for this phenomenon. First, considerable phenotypic and genotypic variation may exist within a single soybean cultivar (Resende et al., 2025); second, morphological divergence may arise from differences in leaf insertion position and developmental stage among leaves of the same variety (Chen et al., 2022). Increased clustering granularity further accentuates intra-cultivar phenotypic heterogeneity.

Further analysis suggests that the mean angle is the core factor driving cluster restructuring and the occurrence of cross-clustering. Both box plot and feature importance results show that the mean angle exhibits the most significant inter-cluster differences and the highest contribution. The variation range of the mean angle within the same variety is significantly higher than that of conventional leaflet shape indices, making it the key factor driving the reassignment of samples. This explains why increasing the number of clusters intensifies cross-clustering; when the clustering granularity is finer, the algorithm prioritizes the division of samples based on the highly variable mean angle, which causes leaves of the same variety to be assigned to different clusters.

Recent manifold-learning phenotyping work by Peng et al. (2026) used t-SNE and UMAP to extract morphological fingerprints of 3D peanut pods for germplasm discrimination (Peng et al., 2026). Their research confirms manifold learning is effective for crop phenotypic stratification, but only utilizes basic size and color global traits, without constructing fine marginal angular metrics. Besides, they did not evaluate trait multicollinearity or intra-variety leaf heterogeneity, the core concerns of our study. Notably, we only adopt t-SNE purely for low-dimensional visualization, rather than as a clustering basis as in their pod-oriented pipeline. Our research focuses on flat soybean leaflets to facilitate field germplasm screening, whereas their work targets underground pods for food traceability. Despite differences in research objects and analytical positioning, both studies prove manifold tools are reliable for resolving morphologically similar samples. Their stratified sampling and cross-validation strategies also offer valuable references for our subsequent multi-regional germplasm validation trials.

The main innovations of this study are as follows: first, this work addresses the limitations of conventional shape-based classification systems by incorporating marginal angular metrics. The newly introduced angle feature exhibits the highest inter-cluster discrimination ability and intra-variety phenotypic variability, which substantially enhances the fine-grained morphological differentiation capacity compared with traditional shape indices alone. Second, this research focuses on the overlooked cross-clustering phenomenon in clustering, clarifying that it is driven by the high variability of angle features, and reveals the inherent laws of phenotypic heterogeneity within soybean varieties.

Nevertheless, the present study focuses solely on static morphological phenotypes without accounting for environmental effects or developmental dynamics. Future research may integrate multi-source phenotyping datasets and controlled experiments to further optimize the classification framework and dissect the underlying mechanisms. Overall, the clustering method based on leaflet shape indices and angle features can achieve the precise classification of soybean leaves. Angle features represent the core factor driving changes in cluster structure and cross-clustering phenomena, while edge complexity acts as a weak discriminative trait that restricts the reliability of manual visual classification. These research results can provide a theoretical basis and technical support for the precise identification of soybean leaflet phenotypes, further investigation into phenotypic heterogeneity, and breeding applications.

## 5 Conclusions

This study investigated the clustering of leaflet morphological phenotypes of 194 soybean varieties. The clustering characteristics of soybean leaves, the causes of cross-clustering phenomena, and the classification validity were clarified by combining t-SNE dimensionality reduction, principal component analysis, cluster validity evaluation, and statistical tests based on Welch’s ANOVA and Games-Howell *post-hoc* comparisons. The following conclusions were drawn:

In the manual classification, the results of t-SNE dimensionality reduction for the three types of soybean leaves demonstrated that two of the three classifications were highly overlapping, with obvious classification confusion stemming from the large number of similar morphological samples for the two types of leaves. Leaflet edge complexity did not differ significantly among these samples, and although the other leaflet shape indices showed significant differences (p < 0.05), the range of variation was small, making it difficult for the naked eye to distinguish them.

The evaluation of clustering validity indicated that when the number of clusters k = 3, the overall clustering quality was better than when k = 4. However, Sankey diagram analysis showed that k = 4 was a reasonable and meaningful refinement of the original K3–1 cluster. The box plot and feature importance heatmap indicated that the angle feature exhibited the most significant distribution difference and the highest contribution rate among the k = 4 clusters, serving as the key indicator for distinguishing the new clusters when k = 4. Statistical tests of the k = 4 clustering results revealed significant pairwise differences for five of the six leaf phenotypic indicators across the four clusters (p < 0.05). Edge complexity was the only trait with non-significant divergence between Group 2 and Group 4, yet the comprehensive morphological signatures of the four clusters remained clearly differentiated. Group 4 could be distinguished by the naked eye, whereas the other three groups were easily confused, but computer clustering could achieve effective distinction relying on multiple combined morphological indicators.

Taken together, the results of this study demonstrate that unsupervised clustering integrating leaflet shape indices and angular features enables precise classification of soybean leaflets. Angular features dominate the refinement of cluster partitioning, while edge complexity constrains the accuracy of manual visual discrimination owing to its weak intergroup discriminatory power. This classification workflow is suitable for high-throughput phenotypic evaluation of breeding progenies and preliminary morphological screening in soybean germplasm banks. Future work should introduce germplasm from multiple ecological regions to further validate the general applicability of this phenotyping pipeline.

## Data Availability

The original contributions presented in the study are included in the article/**Supplementary Material**. Further inquiries can be directed to the corresponding authors.
