# Supplementary material for: Clustering and classification of soybean leaves based on angle features
Source: Front Plant Sci. 2026 Aug 28;17:1908413. doi: 10.3389/fpls.2026.1908413 (PMC13562022; doi:10.3389/fpls.2026.1908413)
Supplement: Supplementary file 1 [file DataSheet1.docx]

Supplementary Material

# 1 Supplementary Data

The full clustering assignment information of all 581 soybean leaflet samples is summarized in Supplementary Table 1. This spreadsheet records the corresponding k3_cluster, k4_cluster and manual_label classification results for each individual terminal leaflet sample, together with unique variety serial numbers and leaf IDs. It fully presents all clustering and manual categorization outcomes for the entire dataset. Readers can directly check the subgroup membership of each leaflet under three classification schemes and track how leaf specimens belonging to the same variety distribute across different clusters, supporting further quantitative analysis of intra-variety leaf morphological heterogeneity and consistency comparison between automated clustering and manual visual classification.

# 2 Supplementary Tables

Levene’s test showed that only eccentricity exhibited homogeneous variance across clusters, whereas the remaining five morphological traits had heterogeneous variances(Supplementary Table 1). Accordingly, Welch’s ANOVA was adopted for subsequent inter-cluster pairwise comparisons to accommodate unequal variances.

Supplementary Table 1. Results of Levene’s test for homogeneity of variances of six soybean leaflet morphological traits

| **Index** | **Abbreviation** | **Levene_F_statistic** | **p_value** | **Variance_Homogeneity** |
| --- | --- | --- | --- | --- |
| Aspect Ratio | AR | 38.527 | 0 | Heterogeneous |
| Eccentricity | Ec | 0.377 | 0.770 | Homogeneous |
| Rectangularity | Re | 3.449 | 0.016 | Heterogeneous |
| Circularity | Ci | 15.031 | 0 | Heterogeneous |
| Edge Complexity | ECo | 12.272 | 0 | Heterogeneous |
| Angle-mean | AM | 33.114 | 0 | Heterogeneous |

Note: Levene’s test was performed to examine the homogeneity of variances prior to one-way ANOVA. Homogeneous variance indicates no significant difference in variance among four clusters (p > 0.05); Heterogeneous variance denotes significant inter-cluster variance difference (p < 0.05).

# 3 Supplementary Tables

Supplementary Code 1. Image processing procedure for soybean leaflet

This Python script implements a complete automated image preprocessing and angular feature extraction pipeline for soybean leaf morphological phenotyping. It first reads grayscale leaf images and leverages Otsu’s algorithm to adaptively compute an optimal segmentation threshold for automatic binarization, eliminating the need for manually fixed cutoff values to separate leaf foreground from complex backgrounds. Subsequent morphological operations including erosion, dilation and small-hole filling are applied to remove noise and repair incomplete leaf contours, followed by extracting the largest connected leaf region and its full outer boundary. The convex hull of the contour points is calculated to identify the primary long axis of the leaf for geometric alignment, and uniform arc-length spline resampling is adopted to standardize contour point density. The pipeline then computes polar angles of evenly sampled marginal points relative to the leaf centroid, sorts these angular values, and finally outputs two core phenotypic outputs: a standardized sequence of marginal polar angles and the mean marginal angle.

Supplementary Code 2. K-Means ARI stability analysis script

This Python script assesses the stability of k = 4 K-means clustering based on six soybean leaf morphological traits. It performs 50 independent K-means runs with single random initializations, calculates the full pairwise Adjusted Rand Index (ARI) matrix via a self-defined ARI function, and computes the mean and standard deviation of upper-triangular ARI values to quantitatively evaluate clustering consistency across repeated iterations. All statistical results and the complete ARI matrix are finally exported to an Excel file.

Supplementary Code 3. Calculation of coefficient of variation and Chi-square variability analysis for soybean leaflet morphological traits

This Python script calculates the coefficient of variation (CV) for six soybean leaf morphological traits across individual varieties, uses the 75% global CV quantile as a threshold to identify varieties with high phenotypic variability for each trait, conducts a global chi-square goodness-of-fit test to examine overall differences in high-variability frequency across traits, and performs pairwise 2×2 contingency table chi-square tests with Bonferroni correction to compare the frequency of high-variability varieties for the mean angle trait against the other five morphological traits, before exporting all frequency statistics and chi-square test results to a structured Excel file.

# 4 Supplementary Figure

Supplementary Figure 1.Visual comparison of leaflet binary images before and after morphological filtering. Representative leaflet binary contours: raw image, image processed with 3×3 morphological opening-closing, and image processed with 5×5 morphological opening-closing. The small kernel sizes effectively eliminate scattered scanning noise while preserving leaf marginal serrations and angular inflection points required for angle-mean feature extraction.
